# Leveraging the Power of Peer Groups for Refugee Integration: A Randomized Field Experiment Comparing Online and Offline Peer Groups

**DOI:** 10.1007/s12599-021-00725-9

**Published:** 2021-11-15

**Authors:** Maximilian Förster, Julia Klier, Mathias Klier, Katharina Schäfer-Siebert, Irina Sigler

**Affiliations:** 1 Institute of Business Analytics, University of Ulm, Helmholtzstraße 22, 89081 Ulm, Germany; 2 Department of Management Information Systems, University of Regensburg, Universitätsstraße 31, 93053 Regensburg, Germany

**Keywords:** Online peer group, Refugee integration, Field experiment, Design science

## Abstract

Refugee integration, one long-term solution to the large number of people fleeing their home countries, constitutes a challenge for both refugees and host societies. ICT and especially online peer groups seem promising to support this process. Building on literature demonstrating the societal benefits of peer groups, this paper proposes a novel peer-group-based approach to address refugee integration and introduces both an online and offline realization. A randomized field experiment in cooperation with public (refugee) services and a non-governmental organization makes it possible to expand existing research by quantitatively demonstrating societal benefits of online peer groups and ICT for refugee integration. Further, this paper is the first to assess the effectiveness of online and offline peer groups in one experimental setup comparatively. Results show that peer groups provide substantial value with respect to the integration domains social bridges, social bonds, rights and citizenship as well as safety and stability. While the outcome of the various integration domains differs for online and offline peer groups, participants’ adoption rates were higher for online peer groups.

**Supplementary Information:**

The online version contains supplementary material available at 10.1007/s12599-021-00725-9.

## Introduction

Humans are born as “ultra-social animals” (Tomasello 2014, p. 187) and started grouping into communities over 50 million years ago (Shultz et al. 2011). Since then, cooperating in groups has been a central strategy for humanity to face challenges. A prominent instrument which builds on this characteristic of human nature are peer groups (Barak et al. 2008). Peer groups differ from other communities (e.g., communities of practice) in such a way that individuals share a need, handicap or desired social/personal change and support each other to overcome their challenging situation or better deal with it (Katz and Bender 1976; Felgenhauer et al. 2019b). Such groups have been proven successful in addressing social problems in various contexts like health (e.g., Cella et al. 1993), career (e.g., Siegel and Donnelly 1978), or racism (e.g., Elligan and Utsey 1999). During the proliferation of the ‘social web’ in the 1990s, a new variant of peer groups emerged: online peer groups (Huber et al. 2018). Indeed, Information and Communication Technology (ICT) can create enormous societal value among geographically dispersed individuals (United Nations 2019) and contribute towards mitigating the consequences of global crises (Thomas et al. 2020), such as supporting refugee integration (Díaz Andrade and Doolin 2016; 2019). Online peer groups have expanded the applicability of peer groups to various social problems and for instance demonstrated positive effects on individuals in the context of unemployment (e.g., Felgenhauer et al. 2019a) and chronic disease (e.g., Wang et al. 2017). What is more, research postulates that ICT might reinforce support in peer groups; still, research calls for extracting the relative importance of online characteristics in online peer groups (Klier et al. 2019).

To the best of our knowledge, no approach exists to date that exploits the potential of online peer groups to effectively enhance refugee integration, one of today’s most pressing issues. The number of refugees, i.e. individuals forcibly displaced due to prosecution, conflict, or general violence, has reached an unprecedented peak of over 25 million worldwide (UNHCR 2020b). Today, integration of this vast number of refugees is a tremendous challenge which confronts both refugees and their host countries. Research indicates that integration of refugees often remains an unsolved issue with refugees risking long-term financial dependency from their host countries, isolation or marginalization as a group, and the hazard of increasing political radicalization in host countries (UNHCR 2013). Even though calls for a “substitute community-type resource” for refugees reach back to the 1980s (Glassman and Skolnik 1984, p. 47), research has rarely dealt with offline peer groups in this context (Badali et al. 2017) and has neglected the societal impact of ICT.

Against this background, we develop a novel peer-group-based approach to enhance refugee integration. We propose a mobile messaging solution (online realization) and a concept for face-to-face meetings (offline realization). Following design science methodology (Hevner et al. 2004), we evaluate the proposed artefact with respect to integration outcomes through a randomized field experiment conducted in cooperation with public (refugee) services and a non-governmental institution. Our contribution to research and practice is threefold. First, we design and implement a novel online peer-group-based approach exploiting the potential of ICT and peer groups in the context of refugee integration. Second, we extend insights into the effects of ICT and online peer groups in the context of refugee integration based on a randomized field experiment, thus answering the call for “more empirically grounded studies” in this context (AbuJarour et al. 2019, p. 15). Third, in a comparative analysis of online and offline peer groups, we quantitatively demonstrate differences in their effectiveness for integration outcomes.

The research presented in this paper is structured as follows: In the next section, we illustrate the problem context and provide an overview of the relevant literature on ICT and peer groups. Afterwards, we propose a novel peer-group-based approach for refugee integration with an online and an offline realization. Then, we demonstrate the practical applicability of our artefact and evaluate its efficacy using a randomized field experiment before we critically discuss implications and limitations of our study and provide directions for further research. Finally, we conclude with a summary of our results.

## Theoretical Background

### Problem Context

The Geneva Convention defines a refugee as an individual who “owing to well-founded fear of being persecuted for reasons of race, religion, nationality, membership of a particular social group or political opinion, is outside the country of his nationality and is unable or, owing to such fear, is unwilling to avail himself of the protection of that country” (UNHCR 1951). The consequences of flight and displacement are severe, not least because in the last decade (2010–2019) merely a fraction of the roughly 100 million people forcibly displaced worldwide could find a solution to their situation (UNHCR 2020a). Thus, local integration of refugees plays a highly relevant role as a durable solution of displacement (UNHCR 2020a).

**Fig. 1:**
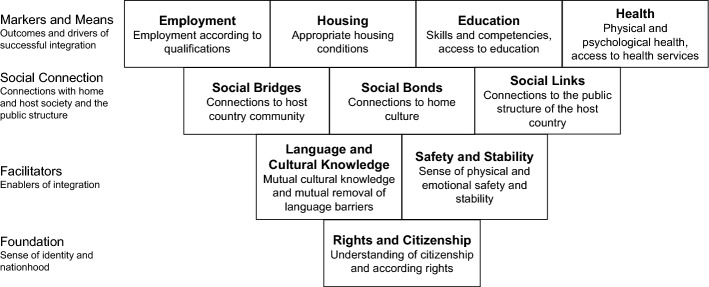
Integration framework by Ager and Strang (2008)

While early scholars equated integration with assimilation into the host society (Park and Burgess 1924), nowadays the UN Refugee Agency describes integration as a concept based on “adaptation” and “welcome” and defines integration along three interlinked dimensions – economic, legal, and social-cultural (UNHCR 2013). Following a modern definition of refugee integration, studies have developed several frameworks and models decomposing the concept of refugee integration into domains or dimensions which show reoccurring key aspects of integration. Harder et al. (2018), for example, differentiate between six dimensions, namely ‘psychological’, ‘economic’, ‘political’, ‘social’, ‘linguistic’, and ‘navigational’. AbuJarour et al. (2018) differentiate between well-being and a sense of agency and, based on a literature review, identify seven dimensions relevant for agency, i.e. ‘social networking’, ‘employment’, ‘education and language’, ‘culture’, ‘health’, ‘government and citizenship’, and ‘housing’. A framework which in great parts corresponds with the framework by AbuJarour et al. (2018) has been proposed by Ager and Strang (2008). This framework is among the most comprehensive models of refugee integration (Hynie et al. 2016) and was developed and verified based on theory and practice, with multiple stakeholders involved (Ager and Strang 2008), and, through its domains, provides “indicators that can be used to evaluate the extent of integration and provide goals for targeting programs” (Hynie et al. 2016, p. 2). Figure 1 shows the ten identified domains related to four overall themes of integration according to Ager and Strang (2008) which serve as a base for the target and evaluation criteria in our study.

Refugee integration is regarded as a dynamic and two-way process, i.e., involving both refugees and host societies (e.g., Da Lomba 2010; Alencar and Tsagkroni 2019). However, the temporal development of integration varies both across different domains of integration and among individuals according to their individual journeys and experiences (Da Lomba 2010). Further, refugees largely differ in their characteristics and background (AbuJarour et al. 2019). This constitutes an important precondition for the design of refugee services and speaks in favour of highly customizable approaches that can be used for support with respect to a broad range of domains of integration.

### ICT for Refugee Integration

Prior research indicates ICT’s potential to help refugees integrating into their host countries (e.g., Siddiquee and Kagan 2006; Bacishoga and Johnston 2013; Díaz Andrade and Doolin 2016; 2019). Mobile phones, for example, have positive effects on social, cultural, and economic participation (Bacishoga and Johnston 2013). Online social networking sites can, for example, serve social connection purposes as well as language and cultural learning purposes (Alencar 2018) and improve women’s access to higher education (Dahya and Dryden-Peterson 2017). Digital services constitute a very promising means of supporting refugees. In recent years, many digital services have been introduced for refugees which address different parts of the refugee journey from predeparture over transit, new arrival, and settling, to longer-term integration (Benton and Glennie 2016). So far, there is a focus on short-term issues of refugee integration, i.e., the first time after arrival. Based on the fact that long-term integration is equally important, there is a call of research focusing on these long-term aspects as well (e.g., AbuJarour et al. 2019).

Prior research has designed and evaluated new approaches aiming to support refugee integration in different aspects. The information platform ‘Integreat’, for instance, offers refugees local information about their municipality by means of different information providers via a mobile application and has been evaluated for optimisation purposes (Schreieck et al. 2017a; 2017b). The mobile application ‘Moin’ features gamification elements and aims at promoting social events for migrant teenagers as well as providing assistance with contextual language learning in Germany (Ngan et al. 2016). While those examples and many other digital services provide refugees with support from host communities, other digital services provide platforms for refugees to help one another. For example, the health services platform ‘New2ukhealth’ was designed to provide peer-to-peer support with respect to health issues in the UK (Benton and Glennie 2016), the question and answer (Q&A) site ‘Wefugees’ provides the opportunity to exchange questions and answers on integration-related topics of all kinds (Schäfer-Siebert and Verhalen 2021), and financial platforms like ‘TransferWise’ or ‘Prosper’ allow for peer-to-peer money transfer or lending (Benton and Glennie 2016). One concept which exploits the potential of mutual support among people sharing the same problem or target, are online peer groups (Katz and Bender 1976). So far, research has neglected to investigate online peer groups as an instrument for enhancing refugee integration. However, research on online peer groups in other contexts suggests a high potential of this concept for the purpose of refugee integration.

### Online Peer Groups and Online Peer Group Effects

Peer groups can be defined as networks of people “who have come together for mutual assistance in satisfying a common need, overcoming a handicap or bringing about desired social and/or personal change” (Katz and Bender 1976, p. 278). People in (online) peer groups have been shown to assist each other in various ways which can be grouped into five types of social support: informational support, emotional support, esteem support, network support, and tangible assistance (Cutrona and Suhr 1992).

Due to the proliferation of digital media, online peer groups have received increasing attention in recent years (Huber et al. 2018). In the realm of online communities, online peer groups focus on users that share a challenging situation and pursue to enhance this situation or how to deal with it through mutual support (Katz and Bender 1976; Felgenhauer et al. 2019b; Bedué et al. 2020). In fact, online peer groups have been proven successful in supporting people facing personal and social challenges in different contexts, first and foremost health-related contexts (e.g., Wang et al. 2017), but also in other contexts like parenting (e.g., Niela-Vilén et al. 2014), employment (e.g., Felgenhauer et al. 2019a), and social isolation among elderly (e.g., Goswami et al. 2010). Peer group effects can be defined as a “change in the belief, attitude or behaviour of a person […] which results from the action or presence [of a peer or group of peers]” (Erchul and Raven 1997, p. 138).

Interest in online peer groups has generated a rich literature in diverse contexts revealing a diversity of positive peer group effects. First, peer groups can foster *knowledge gain* by increasing content knowledge through interaction with peers. For instance, parents in online peer groups report to better understand the role of parenting (Niela-Vilén et al. 2014). Second, peer groups can lead to *positive behaviour change* thus altering detrimental practices. For instance, research indicates that a mobile peer-group-based career counselling approach can significantly increase young people’s chances of finding employment, while improving their career search intensity (Klier et al. 2019). Third, participants of online peer groups can benefit from an *intensification of social connectedness*, which includes feelings of closeness and belonging to peers (Goswami et al. 2010). For instance, elderly people in online peer groups report to escape social exclusion through increased social participation (Goswami et al. 2010). Beyond this, online peer groups can induce intensification of relationships, especially to professional counsellors. Felgenhauer et al. (2019a), for instance, found that unemployed people with complex employment barriers experienced more target-oriented face-to-face employment counselling if at the same time they participated in an online peer group. A fourth positive peer group effect is an *increase of general well-being*. For instance, online peer groups can induce reductions in depression symptoms for women with postpartum depression (Prevatt et al. 2018). Fifth, peer groups have been found to induce an *increase of self-efficacy*, i.e., the “beliefs in one’s capabilities to organise and execute the courses of action required to produce given attainments” (Bandura 1997, p. 3), also referred to as empowerment (Barak et al. 2008) in health-related contexts. For instance, some studies indicate that participation in online peer groups results in improved self-care behaviour of stigmatized chronic diseases (Wang et al. 2017). Apart from those positive effects, some studies also describe unintended side-effects of online peer groups such as the uncritical adoption of potentially harmful information or misinformation (Leist 2013), misuse of personal data (Leist 2013), and harassment under the cloak of anonymity (Cho and Chung 2012).

We expect online peer groups to be an effective means to enhance refugee integration as the five positive peer group effects described above can be directly linked to elements of successful integration (cf. Ager and Strang 2008) and are thus desired outcomes in this context, too. First, refugees need to learn a foreign language and become familiar with a foreign culture (Ager and Strang 2008; OECD/EU 2018). Peer groups might induce this *knowledge gain* (e.g., Niela-Vilén et al. 2014). Second, *positive behaviour change* (e.g., Klier et al. 2019) might contribute to employment, for instance through increased job-search behaviour as could be observed by Klier et al. (2019). Third, *intensification of social connectedness* plays an essential role in integration, as refugees need to keep connections to their home country while building relationships with the people and getting acquainted with the institutions in their host country (Ager and Strang 2008). Online peer groups may foster this connectedness, as they are observed to elevate social participation (e.g., Goswami et al. 2010) and to intensify the relationship to a professional counsellor (Felgenhauer et al. 2019a). Fourth, an *increase of general well-being* (e.g., Prevatt et al. 2018) related to (emotional) safety and stability might be desirable in the context of refugees, as many refugees have experienced violence and persecution. Apart from these parallels between already measured peer group effects in other contexts and domains of successful integration, the peer group effect *increase of self-efficacy* (e.g., Barak et al. 2008) could help refugees along their path of integration. Considering the wide range of challenges for integration, self-reliant coordination between different interventions is indispensable, and a high level of refugees’ self-efficacy might thus contribute to a more target-oriented integration (Desiderio 2016).

To sum up, prior research indicates ICT’s potential to enhance refugee integration. However, there is a scarcity of research on ICT’s potential to assist refugees in integrating into their host countries apart from their first time after arrival. Online peer groups might be promising to enhance refugee integration by means of peer group effects. Despite online peer groups’ striking societal value in various contexts, to date no approach exists that exploits the potential of online peer groups to effectively enhance refugee integration. We aim to address this research gap by conducting a design science study.

## Peer-Group-Based Approach to Enhance Refugee Integration

**Fig. 2:**
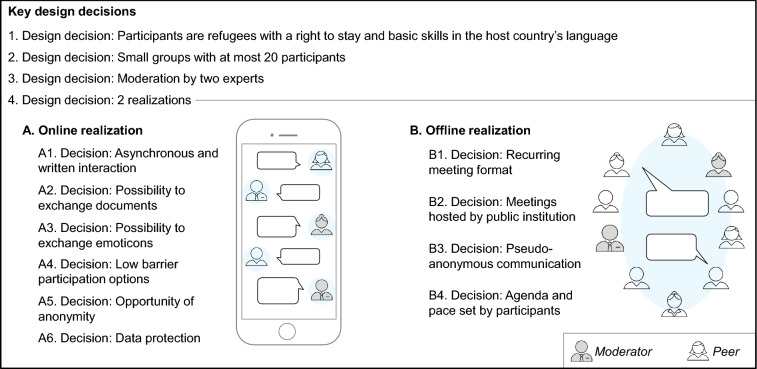
Online peer groups and offline peer groups to enhance refugee integration

In the following, we propose a novel peer-group-based approach to enhance refugee integration. Based on literature, we design two variants of this approach: an online and an offline realization. Both realizations are designed for the refugees (in general) as participants in our approach. The artefact primarily aims at improving refugee integration on behalf of the refugees within the two-way process of refugee integration (cf. e.g., Da Lomba 2010; Alencar and Tsagkroni 2019). However, refugees are also peers, thus representing one central component of our artefact. The peer-group-based approach assists refugees by making use of the enormous potential of peer groups demonstrated in literature. Supplementing existing public and non-governmental interventions, online peer groups (realization A) and offline peer groups (realization B) allow a group of refugees who all need to integrate into a host country to exploit the potential of peer support. In conceptualizing our artefact, we made four major design decisions based on prior research (see Fig. 2).

First, we decided that all refugees with a right to stay and basic skills in the host country’s language qualify as peers, independently of their age, gender, language, or cultural background. Both conditions, i.e., having a right to stay and possessing basic skills in the host country’s language, are linked with a certain duration of stay. This choice of target group is motivated by three main reasons. First, this way, we take up calls for research on phases of integration other than the first time after arrival in the host country (e.g., AbuJarour et al. 2019). Second, this decision ensures that participants share a common challenge (Katz and Bender 1976), i.e., longer-term integration. Consequently, refugees have already gained some experiences in terms of integration challenges, for instance in learning the host country’s language, finding employment, navigating bureaucracy, or identifying leisure activities, and thus might provide mutual understanding and better serve as ‘experts’ for one another (Barak et al. 2008). Finally, the conditions of a right to stay and basic knowledge skills alone still allow for a certain level of heterogeneity within the group which enhances the diversity of knowledge gain and social connectedness within the group (Lyle 2009).

Second, we chose to build small peer groups with each group consisting of at most 20 refugees. This decision is inspired by literature on job clubs suggesting small group sizes (Azrin et al. 1975). Such small group sizes have recently been proven to be effective in the context of job-search among people with complex barriers (Felgenhauer et al. 2019a) and in the context of social support for refugee women (Liamputtong et al. 2016).

Third, we decided that each peer group is moderated by two experts, one professional counsellor from public (refugee) services and one social worker from a non-governmental organization. The moderators’ role is to improve the quality and credibility of information, identified as key design criterion in the refugee setting (Schreieck et al. 2017b), to control the spread of misinformation (e.g., Ross et al. 2018), to prevent bullying (Cho and Chung 2012), and to mediate conflicts that might arise due to cultural tensions (Mogire 2016). Moderators do not introduce any additional pedagogical methods to facilitate improvement along any integration domain in order to allow the peers to determine the way in which the approach is used. The two types of experts allow for a wider range of competencies: While the professional counsellor from public (refugee) services provides expert knowledge on domains such as *employment*, *education* and *language and cultural knowledge*, and existing public interventions addressing other integration domains like *health* and *housing*, the social worker can provide support on a more diverse range of topics including private housing, culture, daily life, mentoring, and social participation. Together, the moderators make it possible to establish *social links* to existing interventions from public services and civil society (cf. Ager and Strang 2008), thereby satisfying the need for coordination and cooperation among actors in the context of refugee integration (Mason and Buchmann 2016).

Finally, we decided to construct a mobile messenger-based variant (online realization) and a face-to-face variant (offline realization) as we expect both variants to offer advantages in our context. The online realization seems particularly beneficial as literature expects ICT and particularly smartphones to substantially facilitate integration (Díaz Andrade and Doolin 2016) and empower refugees (AbuJarour et al. 2021). Also, research indicates high usage of smartphones among refugees (Betts et al. 2017), suggesting that refugees have similar access to mobile networks as the global population (Vernon et al. 2016). More specifically, mobile connectivity is shown to play a critical role during the migration journey (Dekker et al. 2018; Alencar et al. 2019) and in navigating life in Western host countries (Kaufmann 2018), for example by providing access to education (Drolia et al. 2020). Further, non-copresence, enabled through online communication, renders time and location unimportant and allows for access to support from anywhere and at any time (e.g., Coulson 2013). In our context, a refugee might ask for advice on how to negotiate the contract just before viewing a flat and get immediate support from peers in another city who might have already been in the same situation not long ago. However, online communication also entails disadvantages, as copresence, in contrast, helps people to express attitudes, emotions, and positive appraisal thanks to non-verbal expressions (Kiesler et al. 1985). Consequently, participants might feel closer to each other (Sannomiya and Kawaguchi 1999). This is especially beneficial for our target group as social connection is one factor for successful integration (Ager and Strang 2008).

### Online Realization (A)

In conceptualizing the online realization, we built on literature on online communication and online peer groups to arrive at six (sub-)design decisions as functional requirements that allow to best facilitate integration.

First, we designed our application to build on asynchronous and written interaction, with participants primarily communicating via messages. We chose this interaction mode against the backdrop of refugees communicating in a foreign language and discussing also potentially sensitive topics, as it lowers communication barriers (Braithwaite et al. 1999) and gives participants more time to take up utterances (Andresen 2009). Further, this interaction mode grants participants the flexibility to review older information when needed (Bender et al. 2013).

Second, following the example of Klier et al. (2019), our application allows users to exchange documents beyond simple text messages to foster the exchange of information. In our context, information brochures on integration services, or invitations for job-related events, for example, might be shared.

Third, to facilitate the exchange of emotions and to remedy the absence of non-verbal communication, we decided to allow for exchange of emoticons in our application. We built this decision on literature showing that emoticons facilitate the interpretation of text messages (Derks et al. 2008) and even encourage a caring environment (Klier et al. 2019). Also, such visualizations of text are shown to contribute to a feeling of relaxation and closeness in the context of refugee integration (Kaufmann 2018).

Fourth, to mitigate potential difficulties of communicating in written form in a foreign language, we integrated low-barrier participation options that allow taking part in the conversation without having to formulate a text message, such as conducting a poll.

Fifth, we decided to seize the opportunity of anonymity going along with the feature of non-copresence. Definitions of anonymity largely vary in literature, covering for example namelessness or unidentifiability, and have been shown to be related to both positive and negative types of disinhibition like for example self-disclosure or flaming (Lapidot-Lefler and Barak 2012). For our approach, we decided not to use names but anonymous codes for identification in the groups. This namelessness was established to lower the risk of cultural, religious, or gender-related issues. This way, we further account for the fact that anonymity was identified as a desirable feature by research on online peer groups focusing on sensitive issues, like for example communities for former cancer patients (Bender et al. 2013). Apart from the absence of names, participants were free to share personal information about themselves in the chat conversation. This way, we allowed each participant to control their degree of anonymity as research showed that preferences for anonymity also depend on personal characteristics (e.g., Keipi et al. 2015). We aimed to counteract potential negative effects of anonymity through moderators being part of each group.

Sixth, we require the application to fulfil additional safeguards securing data protection to lower the risk of misuse of personal data pointed out by prior literature (Leist 2013) and to meet the requirements of data protection in refugee services (Mason et al. 2017).

Apart from these (sub-)design decisions, non-functional requirements ensure the realization of the functionality (cf. Dabbagh and Lee 2015). First, the mobile messaging application needs to be compatible with standard operating systems to allow low-barrier participation. In our case, the messaging application should be compatible with the standard operating systems iOS and Android to potentially reach as many refugees as possible. Second, as a prerequisite to instantiate and manage small online peer groups, the mobile messaging application needs to allow for the creation of closed groups and the invitation of specific users to those groups.

### Offline Realization (B)

In conceptualizing the offline realization, we built on prior literature on offline communication and face-to-face peer groups to arrive at four (sub-)design decisions that allow to best facilitate integration.

First, we decided for a recurring meeting format aiming to establish a positive routine. This decision was guided by literature on job clubs (Azrin et al. 1975), i.e., a context which is also relevant for refugee integration (Ager and Strang 2008), and by literature on peer groups empowering and improving resilience of refugees (Paloma et al. 2020).

Second, we decided for the partnering public (refugee) institution to host all meetings. This way, we aim to foster the linkage between refugees and offered interventions, another important aspect of integration (Ager and Strang 2008), and to lower participation barriers as potential travel expenses can be reimbursed.

Third, we decided to specify pseudo-anonymous communication in that sharing real names was kept optional and that participants could decide themselves for the amount of personal information they share, like in the online setting. This aims to provide an appropriate level of anonymity and privacy facilitating the discussion of sensitive issues (Bender et al. 2013), especially relevant in the context of refugee integration (Paloma et al. 2020).

Fourth, to keep the approach as customizable as possible, the offline realization also serves merely as a space to facilitate mutual support among peers. Thus, the agenda and pace of the meetings are set by the peers themselves, informed by literature on self-help communities (DeCoster and George 2005).

## Evaluation Strategy

Following design science methodology, we evaluated the utility, quality, and efficacy of our design artefact (Hevner et al. 2004), the peer-group-based approach, and particularly its online and offline realization. We therefore conducted a randomized field experiment and triangled data from three sources to obtain more thorough insights.

### Case Design and Experimental Setting

Conducting a randomized field experiment allowed us to demonstrate the practical applicability of our peer-group-based approach, evaluate its effectiveness and assess online and offline peer groups in the context of refugee integration in a comparative way. The experiment was conducted in cooperation with the German Federal Employment Agency (Bundesagentur für Arbeit) and the German Red Cross at a so-called “Integration Point” in the city of Heidelberg. To respond to a large influx of refugees into Germany since 2015, the Federal Employment Agency instituted “Integration Points” as counselling centres for refugees. The Federal Employment Agency cooperates with municipal authorities and other partners like Employers’ Associations to offer a one-stop shop for refugees in these centres. We chose public services counsellors from the “Integration Point” as moderators for our peer-group-based approach as they possess the required expert knowledge required by our design process. We complemented those moderators through a so-called “integration manager” from the German Red Cross according to our third design decision to include a social worker from a non-governmental organization as moderator in our peer groups. These social workers funded by the state usually guide refugees through the large offer of support services and ensure the provision of knowledge on a more diverse range of integration-related topics which is fundamental to the second kind of moderators in our approach.

We sampled subjects for the pilot study among refugees in both rural and urban districts of the “Integration Point”. According to our design criteria, we focused on refugees with a right to stay in Germany and with German language skills corresponding to the level B1 of the Common European Framework for Languages to ensure that participants in the peer groups could communicate with each other in German. Participation in the experiment was voluntary. Table 1 demonstrates that participants covered a wide range with respect to their duration of stay. On average, they had been living in Germany for roughly three and a half years at the beginning of the experiment.

**Table 1:** Distribution of the participants’ duration of stay

	Min	Max	Median	Mean	Std. Dev
Duration of stay (years)	0.9	9.0	3.4	3.6	0.9

The evaluation of our approach is based on a randomized field experiment with two treatment groups using our peer-group-based approach either realized online (online treatment group, T1) or offline (offline treatment group, T2) and a control group (C) receiving traditional counselling. The experiment was conducted in three phases. In the first phase, five voluntarily participating moderators (four professional counsellors from the Integration Point and one counsellor from the German Red Cross), took part in a four-hour workshop to be introduced into their tasks in the peer-group-based approach aiming to establish a common approach to moderation. Acquisition resulted in 196 refugees deciding to participate in the study, with 65 persons in the online treatment group (T1), 63 persons in the offline treatment group (T2), and 68 in the control group (C). Among the participants, there were 59 women and 137 men aged between 18 and 61 years. Most participants (78%) originally came from Syria. Further countries of origin represented in our sample were Iraq, Somalia, Iran, Eritrea, Russia, Turkey, Afghanistan, and China. We asked all 196 participants to complete a pre-survey. Participants in T1 were assisted in installing and introduced to using the messenger immediately after they had decided to participate in the experiment. Participants in T2 received travel expenses when attending the offline meetings. Thus we aimed to ensure that all participants had access to the respective peer group they were offered. In the second phase (three months), participants received support according to their assignment. In the online treatment group (T1), we connected participants of the online peer groups and their respective moderators via the mobile messaging application “Threema Work” as this application meets all (sub-)design decisions and non-functional requirements (cf. Section 3.1) to make it suitable for our artefact (cf. Table 2). Particularly, it allows for the exchange of text messages, documents, pictures, videos, and emoticons and enables low-barrier participation through conducting polls. In contrast to other well-known mobile messaging applications, it is compliant with the EU General Data Protection Regulation (GDPR) and allows for anonymity by usage of randomized identification numbers for participants and deactivated synchronisation between “Threema Work” contacts and private phone books. Compared to the messaging application “Threema”, which also meets the design requirements, “Threema Work” particularly qualifies for our experiment, as it additionally allows for a central administration of participants’ IDs and the surveillance of their last logins (cf. Section 4.2).

**Table 2:** Exemplary overview of existing messaging applications and fulfilment of requirements

	Threema Work	Threema	Telegram	ginlo	Wire	Signal	WhatsApp
Asynchronous written interaction mode	✓	✓	✓	✓	✓	✓	✓
Possibility to exchange documents	✓	✓	✓	✓	✓	✓	✓
Possibility to exchange emoticons	✓	✓	✓	✓	✓	✓	✓
Low-barrier participation options	✓	✓	✓	✓	✓	✓	✓
Possibility to remain anonymous	✓	✓	✓	✗	✗	✗	✗
Compliance with GDPR	✓	✓	✗	✓	✓	✓	✗
Availability for iOS and Android	✓	✓	✓	✓	✓	✓	✓
Possibility to create closed groups and invite specific users to those groups	✓	✓	✓	✓	✓	✓	✓

**Fig. 3:**
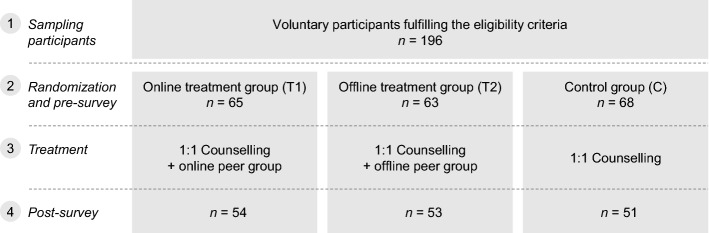
Study design and numbers of participants

In the offline realization of our approach (T2), the weekly one-hour offline meetings of the participants and their moderators were held at the “Integration Point”. The number of groups was chosen such that neither the online peer groups nor the offline meetings exceeded the upper limit of 20 participants determined in our design requirements. The online peer groups and the offline meetings were moderated each by at least one randomly assigned professional counsellor of the “Integration Point” and one social worker from the German Red Cross. The moderators were guided in their moderation tasks by weekly feedback calls and fulfilled the expected role, prevented bullying, added professional knowledge to discussions and shared expert information. Fortunately, there was no need for them to mediate conflicts or to urge participants to be respectful to each other. Online peer groups discussed issues including learning German, finding a job, cultural differences between the home and the host country, leisure activities, and navigating bureaucracy. While these topics were also present in some offline peer group discussions, the latter also included highly intimate topics such as experiences of war and displacement. To help the counsellors in complex situations, we formed a mentoring group using “Threema Work” and instantiated weekly feedback calls with the moderators. In the third phase, we invited all participants again and asked them to complete a post-survey representing the basis for success evaluation. Those who completed the post-survey earned a chance to win regional shopping vouchers worth 15 EUR. We yielded a completion rate of 81% of all 196 participants and counted 54 people in the online treatment group (T1), 53 people in the offline treatment group (T2) and 51 people in the control group (C) who had filled in the pre- and post-survey. Figure 3 summarizes the study design and numbers of participants.

### Data Collection and Measurement

During the experiment, we collected three major datasets: demographic data, usage data, i.e., data on participation in the approach, and survey data.

First, the “Integration Point” provided us with (pseudonymised) demographic data on the participants. This included information on sex, age, country of origin, year of arrival, family status, children, and language level, as these variables have been shown to influence the integration process (Bach et al. 2017). We used this data for robustness purposes.

Second, to capture the adoption of the two realizations of our peer-group-based approach, we gathered data regarding the weekly numbers of participants using the two variants as well as regarding the numbers of participants using the two variants at least once during the three-months period of the experiment. More precisely, to analyse participants’ adoption of the online realization, we collected data on the weekly number of participants using the messenger as well as the number of participants using the messenger at least once during the experiment. This data was gathered by weekly assessing participants’ last login times in the messaging application. To analyse participants’ adoption of the offline realization, we asked the moderators to track the number of attendants for the offline meetings per week as well as the number of participants attending at least one offline meeting.

**Fig. 4:**
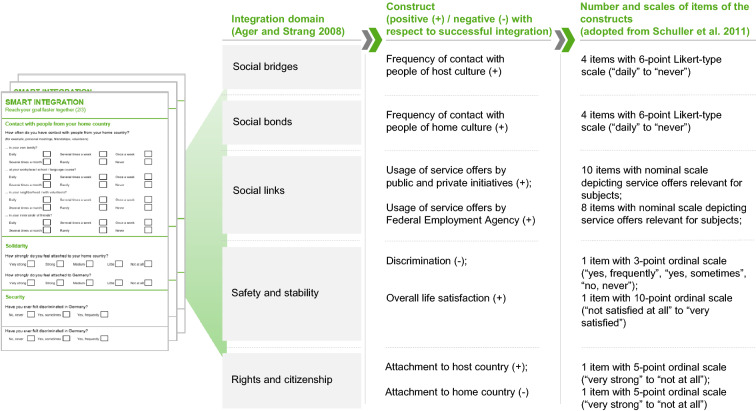
Overview of analyzed constructs measuring success with respect to integration

Third, we measured individual success with respect to the development of integration domains via pre- and post-surveys. In doing so, we follow common practice in research on the success of Information Systems (IS) (cf. Urbach et al. 2009). The surveys captured items which measure successful integration, based on the integration framework by Ager and Strang (2008). To operationalize the domains of integration by Ager und Strang (2008), we mapped constructs from research on the efficacy of another refugee integration intervention in Germany by Schuller et al. (2011) to the integration domains (cf. Figure 4). A more detailed description of the measurements can be found in the appendix (available online via http://link.springer.com).

### Data Analysis

The purpose of our analysis is twofold. First, we analyse the adoption rates of the two realizations of our approach. Second, we assess the efficacy of the online and offline realization of our peer-group-based approach with respect to the constructs measuring integration success described above.

First, to assess the extent to which people take up the offer of the online and offline peer groups, we calculated the average weekly share of participants using the respective realization (average share of participants using the respective realization at least once) and used Chi-square analyses to test for a significant difference between the online and offline peer group. Second, to determine whether there were significant changes in the online treatment group (T1), the offline treatment group (T2), and the control group (C) during the period of observation with respect to the above described constructs on successful integration, we applied the Wilcoxon signed-rank test to the pre and post values of the constructs of each group. As the only systematic difference between T1, T2, and C is the treatment itself, i.e., the implementation of our peer-group-based approach in the online or offline realization, differences in the developments of the groups should be attributable to our approach. Following similar proceedings in IS literature (e.g., Smith et al. 1998; Im and Hars 2001), we chose the Wilcoxon signed-rank test as a non-parametric alternative to the paired-samples t-test because our data was not normally distributed. For handling zeros, the method by Pratt (1959) was used. *P* values were computed based on the conditional null distribution of the test statistic which was approximated by Monte Carlo resampling. To assure comparability of the three groups, i.e., the online treatment group (T1), the offline treatment group (T2) and the control group (C), and thus to make certain that differences between groups result from the experimental manipulation, we verified the random assignment of participants. To do so, we tested for significant differences in characteristics potentially affecting integration recorded in the demographic data. Chi-square analyses on these variables indicated no significant differences between the three groups at the beginning of the experiment.

## Results

### Adoption Rates of the Online and Offline Peer Groups

Our first aim was to analyse whether and to what extent participants in the online and offline treatment groups (T1, T2) took up the approach.

As Table 3 shows, the online peer groups were adopted to a higher extent than the offline peer groups.

**Table 3:** Results on the adoption of the online peer groups (T1) and offline peer groups (T2)

	Number of participants	Number (share) of participants using the approach at least once	Average weekly number (share) of participants using the approach
T1	65	46 (70.8%)	33 (50.8%)
T2	63	37 (58.7%)	7 (11.1%)

More precisely, the share of participants in the online treatment group (T1) who visited the online peer groups at least once (70.8%) was higher than the share of participants assigned to test the offline realization (T2) who attended the offline meetings at least once (58.7%). Furthermore, among those participants in the online treatment group (T1), on average 33 participants (50.8%) logged into the messaging application per week (ranging from 11 to 50 participants across weeks, *SD* = 10). In contrast, in the offline treatment group (T2), the share of participants attending an offline meeting was only 7 participants (11.1%) per week on average (ranging from 0 to 17 participants across weeks, *SD* = 5). A Chi-square test of the difference of average share of participants using the two realizations on a weekly basis indicated high significance (*p* < 0.001). While the average number of participants using the approach on a weekly basis reflects regular usage, it does not capture the intensity of usage (e.g., how many messages were sent or read per participant and how intensively participants took part in the discussions of the offline meetings).

### Efficacy of the Online and Offline Peer Groups with Respect to Refugee Integration

Our second aim was to assess the efficacy of the online and offline realization of our peer-group-based approach with respect to refugee integration, decomposed along the integration domains by Ager and Strang (2008). Table 4 gives an overview of the results.

**Table 4:** Development of groups (T1, T2, C) with respect to constructs measuring integration success

Constructs and related integration domains (positive (+) / negative (−) with respect to successful integration)		Wilcoxon signed-rank test *Z*-statistic (*p < 0.1, **p < 0.05, ***p < 0.01)		
		T1	T2	C
Social bridges (1) Frequency of contact with people of host culture	(+)	–1.98**increase	–1.85** increase	–1.51* increase
Social bonds (1) Frequency of contact with people of home culture	(+)	0.88	–2.28**increase	0.36
Social links (1) Usage of service offers by public and private initiatives	(+)	0.84	0.16	0.51
(2) Usage of service offers by Federal Employment Agency	(+)	1.52* increase	0.61	1.88** increase
Safety and stability (1) Discrimination	(–)	–1.15	–0.69	–1.63* increase
(2) Overall life satisfaction	(+)	–0.58	1.64* increase	–0,35
Rights and citizenship (1) Attachment to host country	(+)	0.65	–2.60*** decrease	–1.46* decrease
(2) Attachment to home country	(–)	−0.49	–0.98	1.76** increase

First, regarding the integration domain social bridges, both the online and the offline treatment groups (T1, T2) significantly improved in the *frequency of contact with people of host culture* (*p* < 0.05). In contrast, the control group (C) only showed an improvement on the 10% significance level. Second, with respect to the domain social bonds, the offline treatment group (T2) showed a significant increase in the *frequency of contact with people of home culture* (*p* < 0.05). In contrast, no significant change in this respect could be detected in the online treatment group (T1) and the control group (C). Third, concerning the domain social links, the control group (C) experienced a significant increase in the *usage of service offers by Federal Employment Agency* on the 5% significance level. While the online treatment group (T1) showed a significant improvement in this respect on the 10% significance level, no such change could be observed in the offline treatment group (T2). Fourth, concerning the domain safety and stability, the control group (C) showed a significant increase in *discrimination* (*p* < 0.1), which could not be observed in the online and offline treatment groups (T1, T2). Further, the offline treatment group (T2) improved significantly with respect to *overall life satisfaction* (*p* < 0.1), whereas the online treatment group (T1) and the control group (C) did not. Finally, regarding the domain rights and citizenship, the control group (C) experienced a significant increase in the *attachment to home country* (*p* < 0.05), while the online and offline treatment groups (T1, T2) did not change significantly. Besides, the control group (C) decreased in the *attachment to host country* on the 10% significance level. Similarly, the offline treatment group (T2) also showed a significant decrease in the *attachment to host country* on the 1% level, whereas the online treatment group (T1) did not show any significant decrease in this respect.

## Discussion

### Implications for Theory and Practice

Following design science methodology, we developed a novel online peer-group-based approach and an offline realization to enhance refugee integration. We implemented both the online and the offline realization of the approach in a randomized field experiment to demonstrate the practical applicability of our approach, to evaluate its effectiveness, and to assess the two realizations in a comparative way. The findings contribute to theory and practice in different ways. From a theoretical point of view, they indicate the following three implications.

First, our study provides strong evidence that peer groups provide substantial value to refugee integration in four of five examined domains of integration by Ager and Strang (2008), i.e., *social bridges*, *social bonds*, *rights and citizenship*, and *safety and stability*. Particularly, our study is the first to establish online peer group effects in the context of refugee integration, by means of a randomized field experiment. First, our study shows that peer groups counteract negative developments in refugees’ attachment to their home and host country which relates to the peer group effect *positive behaviour change*. While the control group showed both a slightly significant decrease in *attachment to host country* (*p* < 0.1) and an (undesired) significant increase in *attachment to home country* (*p* < 0.05), the online peer groups stayed stable in both of these measures. Studies on online peer groups in other contexts found, for example, an enhancement of participants’ attitude towards career choice through online peer groups and eventually their career search intensity (Klier et al. 2019) or positive effects on participants’ physical activity mediated by change in intention (Cavallo et al. 2014). While those changes in attitude are closely linked to behaviour, findings in our study concern a general attitude towards a country. Second, we observe an increase of refugees’ connectedness to the host country community, i.e., non-peers, which relates to the online peer group effect *intensification of social connectedness* (e.g., Goswami et al. 2010; Felgenhauer et al. 2019a). The construct *frequency of contact with people of host culture* significantly increased in online peer groups (*p* < 0.05) compared to only a slightly significant increase in the control group (*p* < 0.1). While former literature shows online peer groups to go along with improved contact with professionals, for example in the context of unemployment (Felgenhauer et al. 2019a), *intensification of social connectedness* in our study refers to people of the host country in general. This peer group effect is highly relevant in the context of refugee integration, as social connectedness both represents a central dimension in several integration frameworks (cf. e.g., Ager and Strang 2008; Hynie et al. 2016; AbuJarour et al. 2018; Harder et al. 2018) and is explicitly referred to as a target indicator for ICT interventions in this context (e.g., AbuJarour et al. 2019). In demonstrating this peer group effect, our approach stands out from existing integration interventions as they are frequently criticized for isolating refugees (Mason and Buchmann 2016).

Second, our findings highlight that online and offline peer groups when established in the same context and in a comparable way are associated with different peer group effects. While online peer groups in our study provided better outcomes in the integration domain *rights and citizenship*, which relates to the peer group effect *positive behaviour change* (e.g., Klier et al. 2019), they showed weaker outcomes in the integration domains *social bonds* and *safety and stability* which relates to the peer group effects *intensification of social connectedness* (e.g., Goswami et al. 2010) and *increase of general well-being* (e.g., Prevatt et al. 2018), respectively. Both online and offline peer groups showed positive outcomes in the domain *social bridges*. To the best of our knowledge, we are the first to quantitatively demonstrate differences in effectiveness between the two foundational realizations of peer groups: online and offline. We thereby extend understanding of ICT impacts by contributing to the so far unanswered research question of the relative importance of online characteristics in peer groups (Klier et al. 2019). In our study, the following differences were apparent between the two realizations: Only online peer groups stayed stable in the construct *attachment to host country*, whereas offline peer groups showed a highly significant, undesired decrease in that measure (*p* < 0.01). In contrast, there was no significant development in online peer groups with respect to *frequency of contact with people of home culture* and *overall life satisfaction*, whereas offline peer groups significantly increased in both variables as desired (*p* < 0.05; *p* < 0.1). Literature on online characteristics and participants’ feedback provides avenues to interpret these differences. Online peer groups are characterized by non-copresence (Coulson 2013). While offline peer groups increased contact with people from their home country, partly by broadening the connection with other refugees in the offline meetings, online peer groups provided support without intensifying contacts amongst each other beyond the participation in the virtual channel. Since online peer group participants only met virtually, they did not strengthen and broaden their network with other refugees, thus, this intervention did not result in increasing their contact to people from their home country. In turn, we conclude that the lower occurrence of a community feeling in the online peer groups allows participants to also feel attached to other people, indicating superior effects with respect to *attachment to host country*. Participants in the offline peer groups reported a different experience with the peer group intervention. They stressed the personal exchange among peers and an atmosphere comparable to a “teahouse”, resulting in a feeling of closeness to peers in offline peer groups in line with literature (Sannomiya and Kawaguchi 1999). Accordingly, prior research suggests that while in online peer groups information plays a more central role, in offline peer groups emotional support and helper therapy are more relevant (Setoyama et al. 2011; Bender et al. 2013). This stronger feeling of connectedness to peers and more central role of helper therapy might explain superior effects of offline peer groups with respect to *frequency of contact with people of home culture* and *overall life satisfaction*.

Through this comparison of online and offline peer groups, we furthermore extend insights into the impact of ICT in the specific context of the study, i.e., refugee integration. Prior studies in this context emphasize the value of ICT with respect to *social bridges* and *social bonds* (e.g., Lloyd and Wilkinson 2017; AbuJarour et al. 2018; Alencar 2018; Kutscher and Kreß 2018). First, while AbuJarour et al. (2018) found that ICT helps resettled refugees to communicate with their friends and family back home and thereby increase their sense of social connectedness, our study suggests that connecting resettled refugees face-to-face is more effective for increasing *social bonds* than connecting them via ICT. Furthermore, existing research proposes that refugees’ online communication with people from the host culture is positively correlated with a sense of social connectedness with people from the host culture (AbuJarour et al. 2018). The results of our study expand these findings and suggest that even online communication among refugees themselves can increase *social bridges*. Thus, online peer groups, although ‘only’ connecting refugees with other refugees, might answer the call for ICT connecting people from the host culture and the home culture (AbuJarour et al. 2019). Finally, prior research found that refugees use ICT to consume and produce cultural content which helps them to maintain a continued connection to their home country (Díaz Andrade and Doolin 2019). In contrast, the online peer groups in our study prove effective for maintaining the *attachment to the host country*: While participation in online peer groups did not increase the *attachment to their home country*, participants in these groups did not experience the decrease of the *attachment to the host country* of the offline peer groups and control group.

Third, our results provide evidence that online peer groups are used to a higher extent than offline peer groups in the context of refugee integration. We find that a significantly higher percentage of participants of the online peer groups (50.8%) used the approach on a regular basis than participants of the offline peer groups (11.1%). While prior research proposes advantages of online peer groups compared to offline peer groups due to time- and location-independent accessibility (Coulson 2013), our study empirically shows that ICT fosters participation in peer groups via a randomized field experiment. In our study, participants reported distance, domestic responsibilities and attending other interventions as main reasons to not make use of the offline peer groups.

Along these theoretical insights, our findings indicate four practical implications to guide decisions in public sector and non-profit organizations.

First, our study demonstrates that peer groups are an effective instrument to enhance refugee integration in four of five dimensions of integration. They particularly help to improve integration by increasing refugees’ social connectedness with people from the home and host country and stabilizing their attachment to the home and host country. Against the background that the latest integration summit in Germany (March 2021) reported mixed results with respect to integration interventions for refugee and migrant integration in Germany over the last 15 years, peer groups represent a highly promising approach for refugee integration.

Second, our results show that there is no one-size-fits-all approach to enhance refugee integration, but rather online and offline peer groups are particularly effective in distinct integration domains. Depending on the specific target of integration, the online or offline realization might thus be more advantageous for public sector organizations and non-profit organizations. Being aware of the differences in effectiveness of the two realizations helps organizations to allocate resources more effectively and efficiently.

Third, in the age of digitalisation, the online realization bears advantages for public sector and non-profit organizations. In particular, the online realization of the peer-group-based approach is more promising for implementation on a larger scale. Indeed, our findings regarding the usage of the two realizations suggest that the online realization provides a low-threshold access for participation via smartphone to the peer group as, on average, online peer groups are used more frequently than offline peer groups. At times of crises like Covid-19, online services often remain the only feasible option. The specific insights into online peer group benefits and effects are becoming more relevant as they support stakeholders of public or social services in quickly and reasonably introducing effective digital services whenever necessary.

Finally, organisations that intend to implement a peer-group-based approach to enhance refugee integration should be aware that online peer groups as a digital service demand different working models and competencies than offline peer groups. To illustrate this, moderators of offline peer groups need to host regular in-person meetings (for instance weekly one-hour meetings as in our study), while moderators of online peer groups can flexibly (in time and location) participate in discussions during working hours. This showcases that digitalisation and digital services go along with different requirements for associated organizations.

### Limitations and Future Research

Aside from the highlighted research contribution presented in this paper, our approach is also subject to limitations which can serve as promising starting points for further research. First, the strengths of our study notwithstanding, our findings are limited regarding the number of participants. Although we could already show significant results for the (separately observed) developments of the two treatment groups and the control group in our study, future research with a larger pool of participants would allow to use more advanced methods to strengthen our results, increase their generality and generate more nuanced insights. For example, methods like differences-in-differences estimators or regression analyses could be used to test for statistical differences between the experimental groups in terms of their development over time. Further, a larger sample would allow for more differentiated insights, e.g., which types of participants extract greater benefit from the online or offline peer groups. Second, the limited observation period of three months did not allow us to analyse long-term effects of our treatments. While we could measure significant developments in domains of integration like *social bonds* and *social bridges* describing refugees’ social connectedness, we for instance only found a mitigating effect in *attachment to host country* for online peer groups and could not investigate all integration domains proposed by Ager and Strang (2008). Still, our research provides a promising starting point for future studies investigating long-term effects of online peer groups for refugee integration. Third, despite the valuable opportunity to conduct a field experiment, the generalizability of our findings might be limited by the fact that we conducted our study in one single setting at one “Integration Point”. Even though Germany hosts the largest absolute number of refugees among EU countries in mid-2020 (UNHCR 2020b), we invite future research to evaluate our peer-group-based approach in other geographical or cultural settings, as studies on ICT in the context of refugee integration are “a context-specific phenomenon” (AbuJarour et al. 2019, p.15). Fourth, in our study, we focused on refugees with basic skills in the home country’s language along with a certain duration of stay to maximize the impact of the (online) peer-group-based approach. However, future studies could design variants of this artefact, which allow also new arrivals to participate and benefit from it, and analyse effects on refugee integration for this target group as well. Fifth, even though our artefact primarily focuses on the refugee perspective of the two-way integration process (cf. e.g., Da Lomba 2010; Alencar and Tsagkroni 2019) both in the design and the evaluation of the artefact, professional counsellors from public (refugee) services and social workers from non-governmental organization take part in the approach as moderators and experts. Through participating in the (online) peer groups, those stakeholders potentially learn from the refugees as well. Consequently, there might be positive effects on the host community through the artefact which could be explored in future research. Sixth, our data collection is based on measurement of constructs’ initial level and final level to determine the subjects’ development in our study. Future research might deepen these insights by observing the continuous development throughout the treatment period, for instance regarding the domain *safety and stability* that may also be subject to more short-term fluctuations. Finally, although we considered two realizations of peer groups for refugees, future studies could conduct another cycle in the iterative design science process (Hevner et al. 2004) and consider further realizations of our artefact, like for example hybrid solutions.

## Conclusion

Peer groups exploit the social element of human nature and provide an approach that builds on the power of peers to face a shared challenge together, both in face-to-face and online settings. Despite abundant evidence demonstrating online peer groups to be successful in addressing social problems in various contexts, to date no approach exists that exploits the potential of online peer groups in the context of refugee integration, one of today’s most pressing issues for both the refugees and their host countries. Further, research calls for assessing the relative importance of ICT in peer groups (Klier et al. 2019).

This study proposed and developed a novel online peer-group-based approach to enhance refugee integration, based on literature on peer groups and ICT effects in peer groups. Besides, we designed an offline realization of the peer-group-based approach. Following design science methodology (Hevner et al. 2004), we evaluated the proposed approach with respect to a well-established framework of integration domains (Ager and Strang 2008) through a randomized field experiment conducted with a unique access at the Federal Employment Agency. Our findings suggest that online peer groups are successful in the integration domains *social bridges, safety and stability*, and *rights and citizenship*. Thus, this research is the first to establish the societal benefits of online peer groups by means of peer group effects in the promising context of refugee integration. Together with promising results for the offline peer groups, we thus provide practitioners with an effective and innovative supplement to existing integration interventions exploiting the power of peers. Further, our findings indicate that in the context of refugee integration, online and offline peer groups provide better outcomes in different domains of integration: While the online peer groups achieved better effects in the domain *rights and citizenship*, the offline peer group achieved better effects in the domains *social bonds* and *safety and stability*. To the best of our knowledge, we were the first to measure and separately examine peer group effects in online and offline peer groups which have been established in a comparable way in the same context. Thereby, we extend existing understanding of ICT impacts in peer groups. We hope our paper will encourage future research to study the fascinating power of online peer groups.

## Supplementary Information

Below is the link to the electronic supplementary material.

Supplementary file1 (PDF 15 kb)
