# Supplementary material for: Leveraging the Power of Peer Groups for Refugee Integration: A Randomized Field Experiment Comparing Online and Offline Peer Groups
Source: Bus Inf Syst Eng. 2021 Nov 15;64(4):441–57. doi: 10.1007/s12599-021-00725-9 (PMC8591321; doi:10.1007/s12599-021-00725-9)
Supplement: Supplementary file 1 — Supplementary file1 (PDF 15 kb) [file 12599_2021_725_MOESM1_ESM.pdf]

Leveraging the Power of Peer Groups for Refugee Integration – A Randomized Field Experiment Comparing Online and Offline Peer Groups

Maximilian Förster, Julia Klier, Mathias Klier, Katharina Schäfer-Siebert, Irina Sigler

Business & Information Systems Engineering (2021)

Appendix (available online via http://link.springer.com)

Constructs Measuring Success with Respect to Integration

We measured success with respect to constructs attributable to the integration domains *social bridges*, *social bonds*, *social links*, *safety and stability*, *language and cultural knowledge* as well as *rights and citizenship* which represent foundation, mediators and facilitators of successful integration (Ager and Strang 2008). As language self-assessment in our questionnaire was misunderstood rather as performance test by participants, we decided to discard the according data. We excluded the domains describing markers and means of integration (i.e. achievements and access across the domains *employment*, *education*, *housing*, *health*) (Ager and Strang 2008). Regarding *housing and health*, this is grounded in the fact that these are elements of primary governmental care, with all member states of the European Union being required to provide accommodation and access to healthcare to refugees (Poptcheva and Stuchlik 2015). *Employment* and *education* on the other hand, require a longer observation period, e.g. considering the duration of an application process, and are influenced by other interventions aiming at achievements in and access to *employment* and *education* such as language courses or vocational training provided by the Federal Employment Agency.

We built measurement on the well-established operationalization of integration measures for Germany (Schuller et al. 2011). Comprehensibility of all survey items was validated with professional counsellors of the "Integration Point". Partially, the language of the constructs was simplified and the constructs referring to the domain *social links* were updated to reflect service offers available at the time of the study. Following the recommendation by Schuller et al. (2011), within the scope of this study, those constructs on successful integration consisting of more than one item were aggregated. The average of a constructs' items was realized for the *frequency of contact with people of host culture* (*social bridges*) and for the *frequency of contact with people of home culture* (*social bonds*); the sum of a constructs' items was realized for the *usage of service offers by public and private initiatives* and for the *usage of service offers by Federal Employment Agency* (*social links*).
